# The association between serum phosphate and length of hospital stay and all-cause mortality in adult patients: a cross-sectional study

**DOI:** 10.1186/s12937-024-00982-w

**Published:** 2024-07-18

**Authors:** Yiquan Zhou, Shuyi Zhang, Zhiqi Chen, Xiaomin Zhang, Yi Feng, Renying Xu

**Affiliations:** 1 Department of Clinical Nutrition, Ren Ji Hospital, Shanghai Jiao Tong University School of Medicine, Shanghai, 200127 China; 2 Intensive Care Medicine, Ren Ji Hospital, Shanghai Jiao Tong University School of Medicine, Shanghai, 200127 China; 3 Department of Clinical Nutrition Center, Xin Hua Hospital, Shanghai Jiao Tong University School of Medicine, Shanghai, 200092 China; 4 Department of Nutrition, College of Health Science and Technology, Shanghai Jiao Tong University School of Medicine, Shanghai, 200025 China

**Keywords:** Hypophosphatemia, Hyperphosphatemia, Length of hospital stay, Mortality, Real-world data

## Abstract

**Background:**

Data is limited on the prevalence of hypophosphatemia in general hospitalized patients, and its association with length of hospital stay (LOS) and mortality remained unclear. We aimed to investigate the prevalence of admission phosphate abnormality and the association between serum phosphate level and length of hospital stay and all-cause mortality in adult patients.

**Methods:**

This was a multi-center retrospective study based on real-world data. Participants were classified into five groups according to serum phosphate level (inorganic phosphorus, iP) within 48 h after admission: **G1**, iP < 0.64 mmol/L; **G2**, iP 0.64–0.8 mmol/L; **G3**, iP 0.8–1.16 mmol/L; **G4**, iP 1.16–1.45 mmol/L; and **G5**, iP ≥ 1.45 mmol/L, respectively. Both LOS and in-hospital mortality were considered as outcomes. Clinical information, including age, sex, primary diagnosis, co-morbidity, and phosphate-metabolism related parameters, were also abstracted from medical records.

**Results:**

A total number of 23,479 adult patients (14,073 males and 9,406 females, aged 57.7 ± 16.8 y) were included in the study. The prevalence of hypophosphatemia was 4.74%. An “L-shaped” non-linear association was determined between serum phosphate level and LOS and the inflection point was 1.16 mmol/L in serum phosphate level. Compared with patients in G4, patients in G1, G2 or G3 were significantly associated with longer LOS after full adjustment of covariates. Each 0.1 mmol/L decrease in serum phosphate level to the left side of the inflection point led to 0.64 days increase in LOS [95% confidence interval (CI): 0.46, 0.81; *p for trend* < 0.001]. But there was no association between serum phosphate and LOS where serum levels of phosphate ≥ 1.16 mmol/L. Multivariable logistic regression analysis showed that adjusted all-cause in-hospital mortality was 3.08-fold greater in patients in G1 than those in G4 (95% CI: 1.52, 6.25; *p for trend* = 0.001). Similarly, no significant association with either LOS or mortality were found in patients in G5, comparing with G4.

**Conclusions:**

Hypophosphatemia, but not hyperphosphatemia, was associated with LOS and all-cause mortality in adult inpatients. It is meaningful to monitor serum levels of phosphate to facilitate early diagnosis and intervention.

**Supplementary Information:**

The online version contains supplementary material available at 10.1186/s12937-024-00982-w.

## Introduction

The prevalence of hypophosphatemia varies dramatically due to different definitions of hypophosphatemia and study populations. Hypophosphatemia occurs in 0.24–12.1% of general hospitalized patients [1, 2]. The prevalence was much higher in patients with advanced cancer [3], acute kidney injury on continuous renal replacement therapy [4–6], and critical illness [7].

Phosphate serves as one of the key elements for energy storage and metabolism in the form of adenosine triphosphate (ATP) [8]. Phosphate abnormalities might lead to multiple organ dysfunction [9], such as impaired myocardial contractility [10], myopathy [11], paresthesia [12], and hematological dysfunction [13]. Considerable interest has been raised in recent years in the relationship between serum phosphate levels and adverse outcomes. Available studies demonstrated a “J-shaped” relationship between serum phosphate level and mortality in critically ill patients [14]. However, most of the studies emphasized the effect of hyperphosphatemia [14–17] and overlooked the effect of hypophosphatemia [18, 19]. One of the possible reasons might lie in that there was no universally accepted definition of hypophosphatemia used in the previous studies [20, 21]. Besides, most of the studies were performed in critically ill patients or patients with hospital-acquired hypophosphatemia (such as refeeding related hypophosphatemia [22] or postoperative hypophosphatemia [23]). Thus, it was uncertain whether hypophosphatemia at admission would lead to longer LOS or increased mortality regardless of the disease severity. As far as we know, only two studies were performed in general hospitalized patients and generated absolutely opposite results from our studies [24, 25].

Thus, we aimed to investigate the association between serum phosphate level within 48 h after admission and clinical outcomes (LOS and in-hospital mortality) in adult inpatients.

## Materials and methods

### Study population

**Fig. 1:**
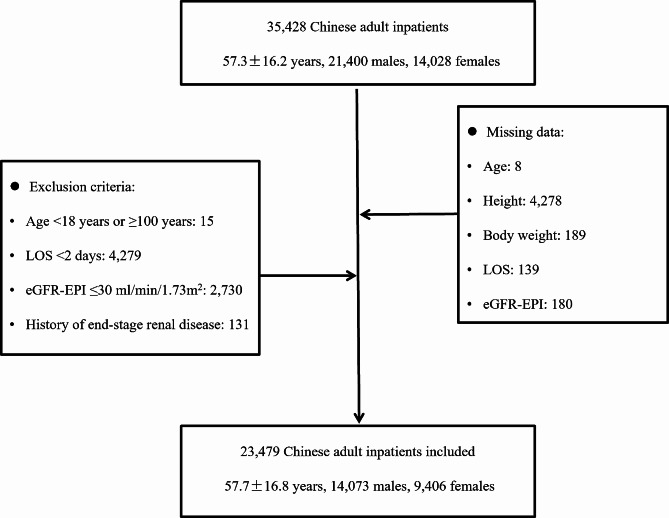
The process of sample recruitment Note: **1.** Abbreviation: **eGFR-EPI**, estimated glomerular filtration rate calculated by Chronic Kidney Disease Epidemiology Collaboration equation; **LOS**, length of hospital stays **2.** History of end-stage of renal disease was confirmed according to admission diagnosis in the medical record

This retrospective study was performed in two teaching hospitals, and both were affiliated to School of Medicine, Shanghai Jiao Tong University in Shanghai, China. All the adult inpatients who were admitted to Ren Ji Hospital from January 1, 2018, to October 31, 2022, or to Xin Hua Hospital from January 1, 2020, to December 31, 2022, and with the availability of serum level of phosphate with 48 h after admission, were included. A total number of 35,428 adult patients were initially recruited. We then performed a sequential process of recruitment: excluding those without information on age (*n* = 8), height (*n* = 4,278), body weight (*n* = 189), LOS (*n* = 139), or estimated glomerular filtration rate (calculated by Chronic Kidney Disease Epidemiology Collaboration equation, eGFR-EPI) (*n* = 180), those who were older than 100 years or younger than 18 years (*n* = 15), those whose LOS < 2 days (*n* = 4,279), or those whose eGFR-EPI < = 30 ml/min/1.73m^2^ (*n* = 2,730), or with the history of end-stage of renal disease according to admission diagnosis (*n* = 131). Finally, a total number of 23,479 patients (14,073 males and 9,406 females, 57.7 ± 16.8 years) were included in the analysis (shown in Fig. 1). Patients included were younger, with a higher rate of surgery, lower Charlson comorbidity index (CCI) [26], lower level of serum phosphate and serum prealbumin, and lower mortality rate than those out of the study (details shown in Supplemental Table 1). The study protocol was approved by the Ethical Committee of Ren Ji Hospital (LY-2022-057-B) and Xin Hua Hospital, (XHEC-C-2023-014-1). As a retrospective study, patients’ written consents were waived.

### Serum level of phosphate (exposure)

All the biochemical parameters were tested within 48 h after admission. Venous blood samples were drawn into vacuum tubes containing Ethylene Diamine Tetraacetic Acid (EDTA) in the morning after participants fasted for at least eight hours as the regular hospital practice in China. Serum level of phosphate was measured by photometric analysis (Roche 701 Bioanalyzer, Roche, UK). The lower limit of detection was 0.1mmol/L. The intra-assay coefficients of variability (CV) were 0.5-0.9% and the inter-assay CV were 1.2-1.9% (Roche 701 Bioanalyzer, Roche, UK).

Patients were further classified into five groups based on serum phosphate level (inorganic phosphorus, iP) [27]: **G1**, iP < 0.64 mmol/L; **G2**, iP 0.64–0.8 mmol/L; **G3**, iP 0.8–1.16 mmol/L; **G4**, iP 1.16–1.45 mmol/L; and **G5**, iP ≥ 1.45 mmol/. Patients in G4 were treated as the reference group.

### Clinical outcomes

LOS was defined as the time between the measurement of serum phosphate and discharge time or the time of death. In-hospital mortality was confirmed based on medical records regardless of the cause of death during hospitalization.

### Assessment of covariates

Baseline characteristics, including age, sex, primary disease for the admission, and comorbidities were abstracted from medical records. CCI without terms of human immunodeficiency virus (HIV) infection and acquired immune deficiency syndrome (AIDS), was used to assess the disease severity because information on HIV infection and AIDS was lacking in the two hospitals. Height and body weight were measured by registered nurses and were abstracted from medical records.

All other laboratory examinations were also abstracted from medical records. Serum levels of calcium, magnesium, sodium, potassium, chloride, liver function test such as alanine aminotransferase (ALT), aspartate aminotransferase (AST), alkaline phosphatase (ALP), gamma glutamyl-transferase (γ-GT), total bilirubin (TBIL), direct bilirubin (DBIL), albumin, and pre-albumin, renal function such as eGFR-EPI, fasting blood glucose (FBG), total cholesterol (TC), triglycerides (TG), low density lipoprotein cholesterol (LDL-C) and high-density lipoprotein cholesterol (HDL-C) were also measured by enzyme linked immunosorbent assay (Roche 701 Bioanalyzer, Roche, UK). White blood cell (WBC) and hemoglobin concentration were measured using an automated hematology analyzer (DxH 690T, Beckman Coulter, USA).

Levels of 25-hydroxyvitamin D [25(OH)D] were determined through an electrochemiluminescence immunoassay, and levels of 25-hydroxyvitamin D_3_ (vitamin D_3_) and 25-hydroxyvitamin D_2_ (vitamin D_2_) were measured by isotope dilution liquid chromatography tandem mass spectrometry. Serum 25(OH)D, vitamin D_2_ and vitamin D_3_ were conducted in Ren Ji Hospital while only 25(OH)D was conducted in Xin Hua Hospital. Low vitamin D status was defined as serum 25(OH)D level < 20 ng/ml [28] or sum of serum vitamin D_3_ and vitamin D_2_ level < 20 ng/ml [29] in the absence of 25(OH)D measurement.

To avoid the effects of hypoalbuminemia on serum level of calcium, albumin-corrected calcium was applied for further analysis, which was calculated as the following equation: serum total calcium (mmol/L) + 0.8×[40-serum albumin (g/L)] [30]. Liver injury was determined as any of the following: ALT (≥120 U/L), AST (≥150 U/L), ALP (≥250 U/L), **γ-**GT (≥100 U/L), or TBIL (≥34.2 μmol/L) [31].

Anemia was determined if serum level of hemoglobin was less than 120 g/L in males or less than 110 g/L in females [32]. Dyslipidemia was determined if one of the following criteria was met: serum TC ≥ 6.2 mmol/L, TG ≥ 2.3 mmol/L, LDL-C ≥ 4.1 mmol/L, or HDL-C < 1.0 mmol/L [33].

### Missing data

In our study, there were 31 (0.1%), 30 (0.1%), 33 (0.1%), 263 (1.1%), 33 (0.1%), 263 (2.2%), 371 (1.6%), 1,311 (5.6%), 109 (0.5%), 1,524 (6.5%), 475 (2%), 859 (3.7%), 1,031 (4.4%), 690 (2.9%), 691 (3%), 707 (5.1%), 676 (2.9) and 264 (0.8%) patients with missing data for ALT, AST, ALP, **γ-**GT, TBIL, DBIL, sodium, calcium, magnesium, chloride, albumin, prealbumin, FBG, TC, TG, LDL-C, HDL-C, WBC and hemoglobin, respectively. Multiple imputations were performed for all the above missing data using chained equation via SPSS in this study. The results with and without multiple imputation were similar (displayed in the Supplemental files). Therefore, we reported the results using origin data.

### Statistics analysis

Continuous variables were tested with the Shapiro-Wilk test for the normality of the distribution and were expressed as the mean±standard deviation (SD) in normal distribution or the median and interquartile range if in abnormal distribution. One-way analysis of variance or the Mann-Whitney U test was performed for comparisons of continuous variables among groups. Categorical variables were described as a number with percentages and were compared using the χ2-test or Fisher exact test.

We performed stepwise regression models to evaluate the association between phosphate categories and LOS. A univariate regression model was analyzed to recruit the risk factors for LOS (Supplemental Table 2), and variables with *p* < 0.1 on univariate analysis were further entering into the multivariate regression model. We used three models to adjust potential confounders. **Model 1**: adjustment of sex and age (“18–45 y”, “45–65 y” ***or*** “≥65 y”); **model 2**: adjustment of variables in **model 1** and further CCI (“0”, “1–2”, ***or*** “≥3”), surgery (“no” vs. “yes”), hospital (Ren Ji Hospital vs. Xin Hua hospital), LOS (adjusted for mortality) (“<7 days”, “7–13 days” ***or***. “≥14 days”), and BMI (“<18.5 kg/m^2^”, “18.5–24 kg/m^2^”, ***or*** “≥24 kg/m^2^”); **model 3**: adjustment of variables in **model 2** and further serum level of eGFR-EPI (“30–60 ml/min/1.73m^2^”, “60–90 ml/min/1.73m^2^”, ***or*** “≥90 ml/min/1.73m^2^”), vitamin D status (“normal” vs. “low”), calcium (“<2.25 mmol/L”, “2.25–2.75 mmol/L”, ***or*** “≥2.75 mmol/L”), magnesium (“<0.75 mmol/L” vs.“≥0.75 mmol/L”), sodium (“<135 mmol/L”, “135–145 mmol/L”, ***or*** “≥145 mmol/L”), chloride (“<96 mmol/L”, “96–108 mmol/L”, ***or*** “≥108 mmol/L”), albumin (“≥35 g/L” vs. “<35 g/L”), pre-albumin (“≥160 mg/dL” vs. “<160 mg/dL”), anemia (“no” vs. “yes”), liver injury (“no” vs. “yes”), dyslipidemia (“no” vs. “yes”), fasting blood glucose (for mortality) (“<3.5mmol/L”, “3.5-7mmol/L”, ***or*** “≥7mmol/L”), and white blood cell count (“<10 × 10^9^/ml” vs. “≥10 × 10^9^/ml”).

We also examined the non-linear relationship between serum phosphate level as continuous variable and LOS using a restricted cubic spline in fully-adjusted model, and confirmed the inflection point if a non-linear relationship existed. Moreover, a two-piecewise linear regression analysis was performed on both sides of the inflection point on the association between each 0.1mmol/L change in serum phosphate level and LOS.

To test the robustness of main results of association between serum phosphate levels and LOS, we performed multiple sensitivity analyses: [1] excluding patients with CCI of 3 points or more (*n* = 3,070) [34]; [2] excluding patients whose eGFR-EPI < 60 ml/1.73m^2^ (*n* = 2,348) [35]; [3] excluding patients whose BMI ≥ 24 kg/m^2^ (*n* = 9,587) or BMI < 18.5 kg/m^2^ (*n* = 1,835) [36, 37]; [4] excluding patients with low vitamin D status (*n* = 15,924) [38]; [5] excluding patients whose hospital LOS ≥ 30 days (*n* = 742); [6] excluding patients whose serum phosphate were not tested within 24 h (*n* = 3,330).

Subgroup analyses were performed for the stratification factors by introducing an interaction term with LOS. Patients were sub-grouped by sex (“male” vs. “female”), age (“18–45 y”, “45–65 y”, ***or*** “≥65”), hospital (Ren Ji Hospital vs. Xin Hua Hospital), CCI (“0”, “1–2”, or “≥3”), serum albumin (“≥35 g/L” vs. “<35 g/L”), prealbumin (“≥160 mg/L” vs. “<160 mg/L”), magnesium (“<0.75 mmol/L” vs. “≥0.75 mmol/L”), and vitamin D status (“normal” vs. “low”).

Moreover, multivariate logistic regression analysis was performed to evaluate the association between serum phosphate categories and in-hospital mortality in fully adjusted model.

All the data were analyzed by SPSS (version 21.0, IBM Corp) and R statistical software tools (http://www.r-project.org, The R Foundation) was used to figure out non-linear relationship. *P* value of < 0.05 was considered as statistical significance.

## Results

### Baseline clinical characteristics of the study population

We finally included a total number of 23,479 patients (14,073 males and 9,406 females, 57.7 ± 16.8 years) in the study. The median of serum phosphate level was 1.14 mmol/L (interquartile range: 1.01 mmol/L, 1.27 mmol/L) and the prevalence of hypophosphatemia was 4.74% (1,112/23,479). Serum level of phosphate was associated with all the baseline characteristics except for ALT (Table 1). The prevalence of phosphate abnormality in patients with different diseases was shown in Supplemental Fig. 1.

**Table 1:** Baseline characteristics of 23,479 Chinese adult inpatients included

Variables	G1	G2	G3	G4	G5	*P* value
iP (mmol/L)	< 0.64	0.64–0.8	0.8–1.16	1.16–1.45	≥ 1.45	
Total, number (%)	341 (1.5)	771 (3.3)	11,479(48.9)	9,241 (39.4)	1,647 (7.0)	< 0.001
Ren Ji Hospital, number (%)	302 (1.5)	657 (3.2)	9,835 (48.5)	7,977 (39.4)	1,489 (7.3)	< 0.001
Xin Hua Hospital, number (%)	39 (1.2)	114 (3.5)	1,644 (51.1)	1,264 (39.3)	158 (4.9)	
Age, y	62.1 ± 17.3	61.7 ± 18.0	60.0 ± 16.6	55.8 ± 16.6	49.2 ± 16.9	< 0.001
Sex, male, %	62.2	74.3	67.3	51	51.7	< 0.001
BMI, kg/m^2^	23.3 ± 3.7	23.0 ± 3.8	23.4 ± 3.7	23.5 ± 3.7	23.4 ± 3.7	0.006
CCI, point	2 (0, 2)	1 (0, 2)	1 (0, 2)	1 (0, 2)	1 (0, 2)	< 0.001
CCI, 0 point, %	36.9	40.6	48.7	38.6	41.2	< 0.001
CCI, 1–2 points, %	46.4	45.1	48.4	49.8	47.8	
CCI ≥ 3 points, %	16.6	14.3	12.3	11.6	11.0	
Surgery, yes, %	58.9	49.0	46.3	46.4	49.6	< 0.001
Mortality, %	5.3	1.9	0.4	0.3	0.5	< 0.001
LOS, day	12, (8, 20)	8, (6, 14)	7, (4, 10)	6, (4, 9)	7, (5, 10)	< 0.001
Phosphate, mmol/L	0.52 (0.40, 0.58)	0.74 (0.70, 0.77)	1.03 (0.95, 1.10)	1.26 (1.17, 1.32)	1.54 (1.49, 1.65)	< 0.001
Calcium, mmol/L	2.12 (2.00, 2.24)	2.18 (2.12, 2.27)	2.21 (2.15, 2.28)	2.23 (2.17, 2.30)	2.25 (2.18, 2.33)	< 0.001
Magnesium, mmol/L	0.84 ± 0.14	0.88 ± 0.10	0.89 ± 0.09	0.90 ± 0.09	0.91 ± 0.10	< 0.001
Sodium, mmol/L	138.7 ± 5.0	139.7 ± 4.2	140.6 ± 3.2	140.7 ± 3.3	140.7 ± 3.3	< 0.001
Potassium, mmol/L	3.5 ± 0.6	3.4 ± 0.5	3.5 ± 0.4	3.6 ± 0.4	3.6 ± 0.5	< 0.001
Chloride, mmol/L	102.8 ± 4.8	103.3 ± 4.5	103.5 ± 4.1	103.3 ± 4.6	102.0 ± 5.1	< 0.001
eGFR-EPI, ml/min/1.73m^2^	97.6 ± 24.9	94.4 ± 26.1	94.9 ± 24.8	97.3 ± 25.4	101.6 ± 27.7	< 0.001
25(OH)D, ng/ml	13.6 (7.6, 18.4)	16.2 (11.1, 23.2)	17.2 (12.3, 23.0)	16.2 (11.6, 21.8)	15.2 (10.8, 20.4)	< 0.001
Vitamin D_2_, ng/ml	0.5 (0.5, 0.9)	0.5 (0.5, 0.7)	0.5 (0.5, 0.8)	0.5 (0.5, 0.8)	0.5 (0.5, 0.7)	< 0.001
Vitamin D_3_, ng/ml	9.0 (5.9, 14.6)	13.4 (8.2, 19.2)	15.3 (10.6, 20.8)	15.1 (10.6, 20.4)	14.7 (10.2, 20.3)	< 0.001
Albumin, g/L	35.4 ± 6.6	37.7 ± 6.3	39.7 ± 5.8	40.0 ± 6.0	40.0 ± 6.7	< 0.001
Pre-albumin, mg/L	159.8 ± 73.5	192 ± 72.4	221.0 ± 60.5	231.3 ± 58.2	231.6 ± 64.7	< 0.001
Hemoglobin, g/L	110.5 ± 31.1	123.6 ± 26.6	130.0 ± 21.9	128.7 ± 20.9	125.3 ± 25.0	< 0.001
ALT, U/L	17, (11, 28)	17, (12, 27)	17, (12, 26)	17, (12, 26)	18, (12, 28)	0.175
AST, U/L	21, (16, 31)	21, (16, 29)	19, (15, 26)	19, (15, 26)	19, (15, 27)	< 0.001
ALP, U/L	75 (61, 99)	77 (64, 96)	75 (61, 91)	74 (60, 90)	73 (60, 90)	< 0.001
γ-GT, U/L	26, (15, 51)	23, (16, 43)	23, (16, 36)	23, (15, 37)	23, (16, 40)	0.003
TBIL, mmol/L	10.8 (7.2, 18.1)	11.0 (7.8, 15.7)	10.4 (7.6, 14.2)	9.6 (7.0, 13.1)	8.8 (6.3, 12.2)	< 0.001
DBIL, mmol/L	3.8 (2.6, 6.4)	3.6 (2.4, 5.3)	3.2 (2.2, 4.5)	2.9 (2.0, 4.0)	2.7 (1.9, 3.8)	< 0.001
TC, mmol/L	3.53 (2.65, 4.48)	3.93 (3.25, 4.79)	4.23 (3.54, 5.02)	4.46 (3.69, 5.28)	4.40 (3.70, 5.33)	< 0.001
TG, mmol/L	1.16 (0.83, 1.72)	1.24 (0.88, 1.78)	1.30 (0.93, 1.85)	1.38 (0.97, 2.02)	1.41 (0.97, 2.19)	< 0.001
FBG, mmol/L	5.7 (4.8, 7.6)	5.5 (4.8, 7.0)	5.4 (4.7, 6.9)	5.3 (4.6, 6.7)	5.2 (4.4, 6.5)	< 0.001
WBC, 10^9^/L	6.7 (4.6, 9.3)	6.2 (5, 8.1)	6.0 (4.9, 7.4)	6.1 (5.0, 7.5)	6.3 (4.9, 7.8)	< 0.001

Note:

1. Abbreviation: **iP**, inorganic phosphorus; **BMI**, body mass index; **CCI**, Charlson Comorbidity index; **LOS**, length of hospital stay; **eGFR-EPI**, estimated glomerular filtration rate calculated by Chronic Kidney Disease Epidemiology Collaboration equation; **ALT**, alanine transferase; **AST**, aspartate aminotransferase; **AKP**, alkaline phosphatase; **γ-GT**, gamma glutamyl-transferase; **WBC**, white blood cell; **FBG**, fasting blood glucose; **HIV**, human immunodeficiency virus; **AIDS**, acquired immune deficiency syndrome; **25(OH)D**, 25 hydroxyvitamin D; **Vitamin D**_**2**_, 25 hydroxyvitamin D_2_; **Vitamin D**_**3**_, 25 hydroxyvitamin D_3_; **TBIL**, total bilirubin; **DBIL**, direct bilirubin; **TC**, total cholesterol; **TG**, triglycerides

2. Abnormal distribution data were shown as median and quartile range

3. CCI without terms of HIV infection and AIDS was used to assess the disease severity

4. LOS was defined as the time between the measurement of serum phosphate and discharge time or the time of death

5. Serum calcium (mmol/L) was calculated as the following equation: serum total calcium (mmol/L) + 0.8×[40-serum albumin (g/L)]

### The association between serum levels of phosphate and LOS

The medium of LOS was 7 days (interquartile range: 4 days, 10 days) in the current study population. An “L-shaped” relationship between serum levels of phosphate and LOS was determined by cubic spine analysis, with a log-likelihood ratio test of *P* < 0.001 (Supplemental Fig. 2). The inflection point of serum phosphate level was 1.16 mmol/L.

Compared with patients in G4, patients in G1, G2 or G3 were significantly associated with longer LOS after adjustment of all potential confounders [G1: β = 4.79, 95% confidence interval (CI): 3.47, 6.10; G2: β = 1.84, 95% CI: 0.94, 2.73; G3: β = 0.44, 95% CI: 0.1, 0.78, respectively]. But no significant association with LOS was found in patients in G5 in the fully-adjusted model (Table 2).

**Table 2:** The association between serum level of phosphate and LOS

Group	iP (mmol/L)	Crude	Model 1	Model 2	Model 3
G1	< 0.64	8.68 (7.35, 10.01)	8.06 (6.73, 9.38)	7.87 (6.56, 9.18)	4.79 (3.47, 6.10)
G2	0.64–0.8	4.10 (3.17, 5.01)	3.40 (2.49, 4.32)	3.30 (2.39, 4.21)	1.84 (0.94, 2.73)
G3	0.8–1.16	1.02 (0.68, 1.36)	0.58 (0.23, 0.93)	0.59 (0.24, 0.93)	0.44 (0.10, 0.78)
G4	1.16–1.45	**0 (ref)**	**0 (ref)**	**0 (ref)**	**0 (ref)**
G5	≥ 1.45	0.41 (-0.24, 1.06)	0.73 (0.08, 1.39)	0.69 (0.05, 1.34)	0.11 (-0.53, 0.74)
*P trend*		< 0.001	< 0.001	< 0.001	< 0.001
Inflection point	1.156	**N/A**	**N/A**	**N/A**	**N/A**
< inflection point	Per 0.1mmol/L decrease	1.14 (0.88, 1.23)	1.05 (0.88, 1.23)	1.02 (0.85, 1.20)	0.64 (0.46, 0.81)
≥inflection point	Per 0.1mmol/L increase	0.02 (-0.04, 0.09)	0.04 (-0.02, 0.1)	0.04 (-0.02, 0.11)	-0.03 (-0.09, 0.03)
*P for log-likelihood ratio test*		< 0.001	< 0.001	< 0.001	< 0.001

Note:

1. Abbreviation: **iP**, inorganic phosphorus; **LOS**, length of hospital stay; **SD**, standard deviation; **BMI**, body mass index; **CCI**, Charlson Comorbidity index; **eGFR-EPI**, estimated glomerular filtration rate calculated by Chronic Kidney Disease Epidemiology Collaboration equation; **25(OH)D**, 25 hydroxyvitamin D; **Vitamin D**_**3**,_ 25 hydroxyvitamin D_3_; **Vitamin D**_**2**,_ 25 hydroxyvitamin D_2_**ALT**, alanine transferase; **AST**, aspartate aminotransferase; **ALP**, alkaline phosphatase; **γ-GT**, gamma glutamyl-transferase; **TBIL**, total bilirubin; **TC**, total cholesterol; **TG**, triglycerides; **LDL-C**, low density lipoprotein cholesterol; **HDL-C**, high density lipoprotein cholesterol; **HIV**, human immunodeficiency virus; **AIDS**, acquired immune deficiency syndrome

2. **Model 1**: adjusting sex and age (“18–45 y”, “45–65 y” ***or*** “≥65 y”)

3. **Model 2**: adjusting variables in **model 1** and further CCI (“0”, “1–2”, ***or*** “≥3”), surgery (“no” vs. “yes”), hospital (Ren Ji Hospital vs. Xin Hua hospital), surgery (“no” vs. “yes”) and BMI (“<18.5 kg/m^2^”, “18.5–24 kg/m^2^”, ***or*** “≥24 kg/m^2^”)

4. **Model 3**: adjusting variables in **model 2** and further serum level of eGFR-EPI (“30–60 ml/min/1.73m^2^”, “60–90 ml/min/1.73m^2^”, ***or*** “≥90 ml/min/1.73m^2^”), vitamin D status (“normal” vs. “low”), calcium (“<2.25 mmol/L”, “2.25–2.75 mmol/L”, ***or*** “≥2.75 mmol/L”), magnesium (“<0.75 mmol/L” vs. “≥0.75 mmol/L”), sodium (“<135 mmol/L”, “135–145 mmol/L”, ***or*** “≥145 mmol/L”), chloride (“<96 mmol/L”, “96–108 mmol/L”, ***or*** “≥108 mmol/L”), albumin (“≥35 g/L” vs. “<35 g/L”), pre-albumin (“≥160 mg/dL” vs. “<160 mg/dL”), anemia (“no” vs. “yes”), liver injury (“no” vs. “yes”), dyslipidemia (“no” vs. “yes”), and white blood cell count (“<10 × 10^9^/ml” vs. “≥10 × 10^9^/ml”)

5. CCI without terms of HIV infection and AIDS was used to assess the disease severity

6. LOS was defined as the time between the measurement of serum phosphate and discharge time or the time of death

7. Serum calcium (mmol/L) was calculated as the following equation: serum total calcium (mmol/L) + 0.8×[40-serum albumin (g/L)]

8. Low vitamin D status was defined as serum 25(OH)D level < 20 ng/ml or sum of serum vitamin D_3_ and vitamin D_2_ level < 20 ng/ml in the absence of 25(OH)D measurement

9. Liver injury was determined as any of the following: ALT (≥120 U/L), AST (≥150 U/L), ALP (≥250 U/L), **γ-**GT (≥100 U/L), or TBIL (≥34.2 μmol/L)

10. Dyslipidemia was determined if one of the following criteria was met: serum TC ≥ 6.2 mmol/L, or TG ≥ 2.3 mmol/L, or LDL-C ≥ 4.1 mmol/L, or HDL-C < 1.0 mmol/L

11. Anemia was determined if serum level of hemoglobin was less than 120 g/L in males, or less than 110 g/L in females

Each 0.1 mmol/L decrease in serum phosphate level to the left side of the inflection point led to 0.64 days increase in LOS (95% CI: 0.46, 0.81; p for trend < 0.001) in a fully-adjusted model. However, no significant difference between each 0.1 mmol/L increase in serum phosphate level and LOS if serum levels of phosphate ≥ 1.16 mmol/L.

Sensitivity analysis generated similar results to the main results (Table 3).

**Table 3:** The association between serum level of phosphate and LOS: sensitivity analysis

Model	G1 (*n* = 341)	G2 (*n* = 771)	G3 (*n* = 11,479)	G4 (*n* = 9,241)	G5 (*n* = 1,647)	Per 0.1mmol/L decrease (iP < 1.16mmol/L)	Per 0.1mmol/L increase (iP ≥ 1.16mmol/L)	*P*_trend_
Sensitivity-1	4.76 (3.38, 6.14)	1.30 (0.36, 2.23)	0.30 (-0.04, 0.65)	**0 (Ref)**	0.24 (-0.40, 0.88)	0.56 (0.37, 0.74)	-0.02 (-0.08, 0.04)	< 0.001
Sensitivity-2	5.42 (4.09, 6.75)	2.01 (1.10, 2.93)	0.51 (0.17, 0.86)	**0 (Ref)**	0.29 (-0.36, 0.94)	0.63 (0.44, 0.82)	-0.03 (-0.08, 0.03)	< 0.001
Sensitivity-3	3.94 (1.88, 5.99)	1.39 (0.02, 2.76)	0.41 (-0.11, 0.94)	**0 (Ref)**	0.09 (-0.87, 1.05)	0.57 (0.29, 0.85)	-0.04 (-0.12, 0.05)	0.002
Sensitivity-4	8.72 (5.96, 11.49)	1.15 (-0.32, 2.62)	0.62 (0.09, 1.16)	**0 (Ref)**	0.48 (-0.58, 1.54)	0.51 (0.23, 0.80)	0.03 (-0.09, 0.19)	< 0.001
Sensitivity-5	2.14 (1.57, 2.71)	1.08 (0.70, 1.45)	0.1 (-0.04, 0.24)	**0 (Ref)**	0.41 (0.15, 0.66)	0.34 (0.32, 0.37)	-0.02 (-0.05, 0.02)	< 0.001
Sensitivity-6	1.88 (0.24, 3.51)	1.15 (0.20, 2.10)	0.36 (0.11, 0.71)	**0 (Ref)**	0.21 (-0.44, 0.87)	0.36 (0.17, 0.56)	0.05 (-0.07, 0.17)	0.008

Note:

1. Abbreviation: **iP**, inorganic phosphorus; **LOS**, length of hospital stay; **SD**, standard deviation; **BMI**, body mass index; **CCI**, Charlson Comorbidity index; **eGFR-EPI**, estimated glomerular filtration rate calculated by Chronic Kidney Disease Epidemiology Collaboration equation; **25(OH)D**, 25 hydroxyvitamin D; **Vitamin D**_**3**,_ 25 hydroxyvitamin D_3_; **Vitamin D**_**2**,_ 25 hydroxyvitamin D_2_; **ALT**, alanine transferase; **AST**, aspartate aminotransferase; **ALP**, alkaline phosphatase; **γ-GT**, gamma glutamyl-transferase; **TBIL**, total bilirubin; **TC**, total cholesterol; **TG**, triglycerides; **LDL-C**, low density lipoprotein cholesterol; **HDL-C**, high density lipoprotein cholesterol; **HIV**, human immunodeficiency virus; **AIDS**, acquired immune deficiency syndrome

2. The model was adjusted by sex, age (“18–45 y”, “45–65 y” ***or*** “≥65 y”), CCI (“0”, “1–2”, ***or*** “≥3”), surgery (“no” vs. “yes”), hospital (Ren Ji Hospital vs. Xin Hua hospital), BMI (“<18.5 kg/m^2^”, “18.5–24 kg/m^2^”, ***or*** “≥24 kg/m^2^”), serum level of eGFR-EPI (“30–60 ml/min/1.73m^2^”, “60–90 ml/min/1.73m^2^”, ***or*** “≥90 ml/min/1.73m^2^”), vitamin D status (“normal” vs. “low”), calcium (“<2.25 mmol/L”, “2.25–2.75 mmol/L”, ***or*** “≥2.75 mmol/L”), magnesium (“<0.75 mmol/L” vs. “≥0.75 mmol/L”), sodium (“<135 mmol/L”, “135–145 mmol/L”, ***or*** “≥145 mmol/L”), chloride (“<96 mmol/L”, “96–108 mmol/L”, ***or*** “≥108 mmol/L”), albumin (“≥35 g/L” vs. “<35 g/L”), pre-albumin (“≥160 mg/dL” vs. “<160 mg/dL”), anemia (“no” vs. “yes”), liver injury (“no” vs. “yes”), dyslipidemia (“no” vs. “yes”), and white blood cell count (“<10 × 10^9^/ml” vs. “≥10 × 10^9^/ml”)

3. CCI without terms of HIV infection and AIDS was used to assess the disease severity

4. LOS was defined as the time between the measurement of serum phosphate and discharge time or the time of death

5. Serum calcium (mmol/L) was calculated as the following equation: serum total calcium (mmol/L) + 0.8×[40-serum albumin (g/L)]

6. Low vitamin D status was defined as serum 25(OH)D level < 20 ng/ml or sum of serum vitamin D3 and vitamin D_2_ level < 20 ng/ml in the absence of 25(OH)D measurement

7. Liver injury was determined as any of the following: ALT (≥120 U/L), AST (≥150 U/L), ALP (≥250 U/L), **γ-**GT (≥100 U/L), or TBIL (≥34.2 μmol/L)

8. Dyslipidemia was determined if one of the following criteria was met: serum TC ≥ 6.2 mmol/L, or TG ≥ 2.3 mmol/L, or LDL-C ≥ 4.1 mmol/L, or HDL-C < 1.0 mmol/L

9. Anemia was determined if serum level of hemoglobin was less than 120 g/L in males, or less than 110 g/L in females

10. Sensitivity-1: excluding patients whose CCI ≥ 3 points (*n* = 3,070)

11. Sensitivity-2: excluding patients whose eGFR-EPI < 60 ml/min/1.73m^2^ (*n* = 2,348)

12. Sensitivity-3: excluding patients whose body mass index ≥ 24 kg/m^2^ (*n* = 9,587) or < 18.5 kg/m^2^ (*n* = 1,835)

13. Sensitivity-4: excluding patients with low vitamin D status (*n* = 15,924)

14. Sensitivity-5: excluding patients whose LOS ≥ 30 days (*n* = 742)

15. Sensitivity-6: excluding patients whose serum phosphate were not tested within 24 h (*n* = 3,330)

Subgroup analysis demonstrated that the association between hypophosphatemia and LOS was stronger among elderly patients older than 65 years and patients with severe comorbid condition (CCI ≥ 3). Such significant association was only found in patients with normal albumin or prealbumin concentration (Supplemental Table 3).

### The association between serum levels of phosphate and in-hospital mortality

The prevalence of all-cause mortality was 0.5% (111/23,479). Multivariable logistic regression analysis showed that adjusted all-cause in-hospital mortality was 3.08-fold greater in patients in G1 than those in G4 [odd ratio (OR) 95% CI: 1.52, 6.25; *p for trend* = 0.001] (Table 4). Furthermore, each 0.1 mmol/L decrease in serum phosphate increased the risk of in-hospital mortality by 11% (adjusted OR = 1.11, 95% CI: 1.03, 1.2). However, hyperphosphatemia was not significantly associated with in-hospital mortality (Table 4).

**Table 4:** The association between serum level of phosphate and in-hospital mortality: multivariate logistic regression

Model	G1 (*n* = 341)	G2 (*n* = 771)	G3 (*n* = 11,479)	G4 (*n* = 9,241)	G5 (*n* = 1,647)	Per 0.1mmol/L decrease	*P*_trend_
Death	18	15	41	29	8	**N/A**	**N/A**
Mortality, %	5.28	1.95	0.36	0.31	0.53	**N/A**	**N/A**
All-adjusted model	3.08 (1.52, 6.25)	1.73 (0.86, 3.46)	0.80 (0.49, 1.33)	1 (Ref)	1.22 (0.52, 2.84)	1.11 (1.03, 1.20)	0.011

Note:

1. Abbreviation: **iP**, inorganic phosphorus; **LOS**, length of hospital stay; **SD**, standard deviation; **BMI**, body mass index; **CCI**, Charlson Comorbidity index; **eGFR-EPI**, estimated glomerular filtration rate calculated by Chronic Kidney Disease Epidemiology Collaboration equation; **25(OH)D**, 25 hydroxyvitamin D; **Vitamin D**_**3**,_ 25 hydroxyvitamin D_3_; **Vitamin D**_**2**,_ 25 hydroxyvitamin D_2_; **ALT**, alanine transferase; **AST**, aspartate aminotransferase; **ALP**, alkaline phosphatase; **γ-GT**, gamma glutamyl-transferase; **TBIL**, total bilirubin; **TC**, total cholesterol; **TG**, triglycerides; **LDL-C**, low density lipoprotein cholesterol; **HDL-C**, high density lipoprotein cholesterol; **HIV**, human immunodeficiency virus; **AIDS**, acquired immune deficiency syndrome

2. The model was adjusted by sex, age (“18–45 y”, “45–65 y” ***or*** “≥65 y”), CCI (“0”, “1–2”, ***or*** “≥3”), surgery (“no” vs. “yes”), hospital (Ren Ji Hospital vs. Xin Hua hospital), LOS (“<7 days”, “7–13 days” ***or***. “≥14 days”), BMI (“<18.5 kg/m^2^”, “18.5–24 kg/m^2^”, ***or*** “≥24 kg/m^2^”), serum level of eGFR-EPI (“30–60 ml/min/1.73m^2^”, “60–90 ml/min/1.73m^2^”, ***or*** “≥90 ml/min/1.73m^2^”), vitamin D status (“normal” vs. “low”), calcium (“<2.25 mmol/L”, “2.25–2.75 mmol/L”, ***or*** “≥2.75 mmol/L”), magnesium (“<0.75 mmol/L” vs. “≥0.75 mmol/L”), sodium (“<135 mmol/L”, “135–145 mmol/L”, ***or*** “≥145 mmol/L”), chloride (“<96 mmol/L”, “96–108 mmol/L”, ***or*** “≥108 mmol/L”), albumin (“≥35 g/L” vs. “<35 g/L”), pre-albumin (“≥160 mg/dL” vs. “<160 mg/dL”), anemia (“no” vs. “yes”), liver injury (“no” vs. “yes”), dyslipidemia (“no” vs. “yes”), fasting blood glucose (“<3.5 mmol/L”, “3.5-7 mmol/L”, ***or*** “≥7 mmol/L”), and white blood cell count (“<10 × 10^9^/ml” vs. “≥10 × 10^9^/ml”)

3. CCI without terms of HIV infection and AIDS was used to assess the disease severity

4. LOS was defined as the time between the measurement of serum phosphate and discharge time or the time of death

5. Serum calcium (mmol/L) was calculated as the following equation: serum total calcium (mmol/L) + 0.8×[40-serum albumin (g/L)]

6. Low vitamin D status was defined as serum 25(OH)D level < 20 ng/ml or sum of serum vitamin D_3_ and vitamin D_2_ level < 20 ng/ml in the absence of 25(OH)D measurement

7. Liver injury was determined as any of the following: ALT (≥120 U/L), AST (≥150 U/L), ALP (≥250 U/L), **γ-**GT (≥100 U/L), or TBIL (≥34.2 μmol/L)

8. Dyslipidemia was determined if one of the following criteria was met: serum TC ≥ 6.2 mmol/L, or TG ≥ 2.3 mmol/L, or LDL-C ≥ 4.1 mmol/L, or HDL-C < 1.0 mmol/L

9. Anemia was determined if serum level of hemoglobin was less than 120 g/L in males, or less than 110 g/L in females

## Discussion

In this current retrospective study based on real-world data, the prevalence of hypophosphatemia and hyperphosphatemia were 4.74% and 7.01%, respectively. An “L-shaped” non-linear relationship between admission phosphate levels and LOS was demonstrated with the inflection point of 1.16mmol/L. Serum phosphate level < 1.16 mmol/L, but not hyperphosphatemia, was significantly associated with prolonged LOS after adjustment of age, sex, CCI, and other potential confounders. Each 0.1 mmol/L decrease in serum phosphate when iP < 1.16mmol/L could lead to 0.64 days increase in LOS regardless of disease severity. Besides, longer LOS could be observed in older patients with moderate-to-severe hypophosphatemia with severe comorbid condition (CCI ≥ 3 points) in the subgroup analysis, which could be partly explained by subsequent malnutrition [39], tubular dysfunction [40] and inflammation [39]. Interestingly, significant association between hypophosphatemia and prolonged LOS was only found in patients with normal albumin or prealbumin concentration, which brought forth the proposal of screen for hypophosphatemia in well-fed patients. Furthermore, only admission moderate-to-severe hypophosphatemia was an independent risk factor for all-cause mortality in adult patients in fully-adjusted model.

### Main findings in previous studies

We found that limited studies have been focusing on the association between admission hypophosphatemia and adverse outcomes in general inpatients regardless of disease severity, as most of studies were performed in critically ill patients.

To date, most of previous studies addressed that hyperphosphatemia at admission could be an independent risk factor for mortality in critical illness [14, 15, 17, 18, 41–43]. On the contrary, the association between hypophosphatemia and clinical outcome was inconsistent in recent studies. Broman et al. [44] conducted a large retrospective observational study including 4,656 patients at combined ICU in Sweden and found out no significant association could be observed between hypophosphatemia and ICU mortality or hospital mortality, even taking different criteria of hypophosphatemia (iP < 0.5, 0.3, or 0.2 mmol/L, respectively) and the duration of hypophosphatemia into account, which was consistent with a recent meta-analysis including 12 studies with 7,626 ICU patients [21] and other studies with large sample size [7, 45–47]. Meanwhile, Wang et al. [48] studied 946 general ICU patients in China and reported that hypophosphatemia was an independent risk factor for 28-day ICU mortality (adjusted OR = 1.5, 95% CI: 1.1, 2.1, *p* = 0.01), which was consistent with the results from another retrospective cohort including 13,155 ICU patients [16]. Another study on 9,691 sepsis patients from MIMIC-IV cohort brought out the unique result that hypophosphatemia on the second day might be independently associated with reduced 28-day mortality and act as a protective factor [41]. Furthermore, several studies demonstrated significant associations between hypophosphatemia and prolonged LOS in hospital [21] or in ICU [21, 44], but few didn’t [47].

As far as we know, only two studies were performed in general hospitalized patients and generated the results quite different from our study. Both of studies demonstrated a “J-shaped” relationship between admission phosphate and in-hospital mortality [24, 25]. One study including 42,336 adult inpatients admitted to Mayo Clinic between 2009 and 2013, patients with hyperphosphatemia (G6: iP≥1.52mmol/L) had a higher risk of death than those with hypophosphatemia (G1: iP < 0.78mmol/L) (G1: OR = 1.6, 95%CI, 1.25 to 2.05; G6: OR = 3.89, 95%CI, 3.2 to 4.74, respectively) compared with patients with normophosphatemia (G3: iP 1-1.16mmol/L) [24]. Another study performed in inpatients with infectious diseases showed that only hyperphosphatemia was associated with a slight increase of 0.33 days in LOS but not mortality, and no significant association between hypophosphatemia and mortality could be observed [25].

### Possible reasons for difference between our research and previous studies

 [1]. Varied definitions of phosphate abnormality: the cut-off value for hypophosphatemia varied from 0.6 to 1.09 mmol/L [7, 14, 15, 18, 24, 44], and the cut-off value for hyperphosphatemia varied from 1.23 to 1.94mmol/L [15, 18, 24, 44, 49]. Besides, some studies used the lowest or highest quartile of serum phosphate as the low or high phosphate level [50]. As normal serum phosphate levels ranged from 0.8 to 1.45mmol/L in adults and inflection point of 1.16mmol/L in serum phosphate level was observed in our study, hypophosphatemia and hyperphosphatemia were defined as serum phosphate level < 0.8mmol/L (G1 and G2) and ≥1.45mmol/L (G5), respectively, and G4 (iP 1.16-1.45mmol/L) was regarded as reference.

 [2]. Different study populations: as most of the previous studies were performed in critically ill patients [7, 16, 44, 48], or trauma patients [50] or sepsis patients [14, 18, 49], the effects of admission hypophosphatemia on mortality or other adverse outcomes could be easily concealed or eliminated by the severity of diseases.

 [3]. Severity of hypophosphatemia: we categorized patients into 5 groups according to serum levels of phosphate, among which G1 was regarded as moderate-to-severe hypophosphate (iP < 0.64 mmol/L) and G2 was regarded as mild hypophosphate (iP 0.64–0.8 mmol/L) [27]. Compared to G4 (iP 1.16–1.45 mmol/L), serum phosphate level < 1.16 mmol/L was significantly associated with longer LOS. Although the prevalence of moderate-to-severe hypophosphatemia was only 1.5%, it acted as the strongest predictor for risk of death and prolonged LOS regardless of disease severity. The reason for such phosphate abnormality included short-term diet restriction, malnutrition, and disease severity.

 [4]. Exclusion of patients with impaired renal function: as serum phosphate levels were shown as non-linear association with eGFR-EPI with the inflecion point as 30 ml/min/1.73m^2^ in a Japanese cohort [35], serum phosphate level was negatively associated with eGFR-EPI when it below 30 ml/min/1.73m^2^. Considering the mixed effect of hormone change, medication or RRT along with the impaired renal function on the association between serum phosphate and adverse outcome, we excluded those patients with eGFR-EPI < 30 ml/1.73m^2^ or history of end-stage renal disease, therefore the number of patients with hyperphosphatemia may decrease and the adverse effect of hyperphosphatemia could be underestimated. The reason for different conclusion between our study and previous studies performed in general inpatients may partly lie in that they didn’t exclude patients with impaired renal function, so that the effects of hyperphosphatemia on adverse clinical outcomes could be observed.

### Strengths and limitation of our study

The strengths of our study included that it was a multi-center retrospective cohort study based on real-world data which could ensure a large sample size, with a full adjustment of many important confounders including phosphate metabolism-related parameters (serum level of calcium and vitamin D) and severity of disease (CCI). More importantly, we offered the evidence that maintaining upper-to-normal-limit of serum phosphate may help to lower the probability of adverse outcomes such as prolonged LOS or risk of death in hospital. However, our study had several limitations. Firstly, the design of retrospective cross-sectional study limited the evidence level. Besides, missing data were common because of real-world data extracted from the electronic medical record system. Excluding those with missing data might lead to selection bias. Secondly, exclusion of patients whose eGFR-EPI < 30 ml/1.73m^2^ or those with history of end-stage renal disease would decrease the number of patients with hyperphosphatemia and underestimate its adverse effect on mortality and LOS. Thirdly, there was still a lack of information on several elements in the complex interplay linking serum phosphate level and mortality such as Corona Virus Disease 2019 (COVID-19) [51] and phosphate-regulating hormones including parathyroid hormone (PTH) and fibroblast growth factor-23 (FGF-23) [20]. Finally, we didn’t collect the information regarding the etiology of phosphate abnormalities (such as dietary intake before hospitalization, gastrointestinal symptoms, proteinuria, body composition related to phosphorus distribution, or medication affecting phosphate metabolism), patterns of phosphate abnormalities (chronic, acute or transient), intervention of phosphate abnormalities and changes in serum phosphate during hospitalization. A well-designed prospective study on the association between individualized phosphate repletion therapy or phosphorus fluctuation model and outcome was necessary to duplicate our results.

## Conclusions

The overall prevalence of hypophosphatemia was 4.74%. Moderate-to-severe hypophosphatemia, but not hyperphosphatemia, was an independent risk factor for prolonged LOS and all-cause mortality regardless of disease severity. Regular monitoring and maintaining an optimal range of serum phosphate levels might be helpful for inpatients to reduce the risk of bad clinical outcomes.

### Electronic supplementary material

Below is the link to the electronic supplementary material.

Supplementary Material 1

## Data Availability

No datasets were generated or analysed during the current study.

## Abbreviations

γ-GT: Gamma glutamyl-transferase
25(OH)D: 25-hydroxyvitamin D
AIDS: Acquired immune deficiency syndrome
ALP: Alkaline phosphatase
ALT: Alanine aminotransferase
AST: Aspartate aminotransferase
ATP: Adenosine triphosphate
BMI: Body mass index
CCI: Charlson comorbidity index
CI: Confidence interval
CV: Coefficients of variability
D_3_: 25-hydroxyvitamin D_3_
EDTA: Ethylene Diamine Tetraacetic Acid
eGFR-EPI: Calculated by Chronic Kidney Disease Epidemiology Collaboration equation
FBG: Fasting blood glucose
FGF-23: Fibroblast growth factor-23
HDL-C: High-density lipoprotein cholesterol
HIV: Human immunodeficiency virus
ICU: Intensive care unit
iP: Inorganic phosphorus
LDL-C: Low density lipoprotein cholesterol
LOS: Length of hospital stay
OR: Odd ratio
PTH: Parathyroid hormone
SD: Standard deviation
TBIL: Total bilirubin
TC: Total cholesterol
TG: Triglycerides
