# Supplementary material for: The association between serum phosphate and length of hospital stay and all-cause mortality in adult patients: a cross-sectional study
Source: Nutr J. 2024 Jul 18;23:81. doi: 10.1186/s12937-024-00982-w (PMC11256428; doi:10.1186/s12937-024-00982-w)
Supplement: Supplementary file 1 — Supplementary Material 1 [file 12937_2024_982_MOESM1_ESM.docx]

**Supplemental Table 1**. Baseline characteristics between hospitalized patients in inclusion and exclusion groups

| Variables | In the study (n=23,479) | Out of the study (n=11,949) | P value |
| --- | --- | --- | --- |
| Age, y | 57.7±16.9 | 58.1±16.2 | 0.048 |
| Sex, male, % | 59.9 | 61.3 | 0.012 |
| Body mass index, kg/m^2^ | 23.4±3.7 | 23.5±3.7 | 0.188 |
| CCI, point | 1 (0, 2) | 2 (0, 3) | <0.001 |
| Surgery, yes, % | 46.8 | 31.8 | <0.001 |
| LOS, day | 7 (4, 10) | 3 (1, 8) | <0.001 |
| In-hospital mortality, % | 0.5 | 12.1 | <0.001 |
| Phosphate, mmol/L | 1.14 (1.01, 1.27) | 1.20 (1.04, 1.42) | <0.001 |
| Calcium, mmol/L | 2.22 (2.16, 2.29) | 2.21 (2.13, 2.30) | <0.001 |
| Magnesium, mmol/L | 0.89±0.09 | 0.92±0.12 | <0.001 |
| Potassium, mmol/L | 3.57±0.42 | 3.77±0.59 | <0.001 |
| Sodium, mmol/L | 140.6±3.3 | 140.4±3.6 | <0.001 |
| Chlorine, mmol/L | 103.6 (101.5, 105.7) | 103.1 (100.8, 105.4) | <0.001 |
| eGFR-EPI, ml/min/1.73m^2^ | 98.5 (81.9, 112.8) | 80.8 (15.9, 103.8) | <0.001 |
| 25(OH)D, ng/ml | 16.5 (11.8, 22.2) | 14.7 (9.6, 20.8) | <0.001 |
| vitamin D_3_, ng/ml | 15.0 (10.4, 20.5) | 14.3 (9.8, 19.8) | <0.001 |
| Albumin, g/L | 40.5 (37.1, 43.4) | 40.1 (35.6, 43.5) | <0.001 |
| Pre-albumin, mg/L | 224.3±61.5 | 253.7±76.3 | <0.001 |
| Liver injury, yes, % | 5.6 | 4.5 | <0.001 |
| Dyslipidemia, yes, % | 48.0 | 51.4 | <0.001 |

**Note:**

1. Abbreviation: **CCI**, Charlson Comorbidity index; **LOS**, length of hospital stay; **eGFR-EPI**, estimated glomerular filtration rate calculated by Chronic Kidney Disease Epidemiology Collaboration equation; **ALT**, alanine transferase; **AST**, aspartate aminotransferase; **AKP**, alkaline phosphatase; **γ-GT**, gamma glutamyl-transferase; **WBC**, white blood cell; **HIV,** human immunodeficiency virus; **AIDS**, acquired immune deficiency syndrome, **25(OH)D**, 25 hydroxyvitamin D; vitamin **D_2_**, 25 hydroxyvitamin D_2_; vitamin **D_3_**, 25 hydroxyvitamin D_3_.
2. Abnormal distribution data was shown as median and quartile range.

3. CCI without terms of HIV infection and AIDS was used to assess the disease severity

4. LOS was defined as the time between the measurement of serum phosphate and discharge time or the time of death.

5. Serum calcium (mmol/L) was calculated as the following equation: serum total calcium (mmol/L) + 0.8×[40-serum albumin (g/L)].

6. Liver injury was determined as any of the following: ALT (≥120 U/L), AST (≥150 U/L), ALP (≥250 U/L), **γ-**GT (≥100 U/L), or TBIL (≥34.2 μmol/L).

7. Dyslipidemia was determined if one of the following criteria was met: serum TC ≥6.2 mmol/L, or TG≥2.3 mmol/L, or LDL-C≥4.1 mmol/L, or HDL-C<1.0 mmol/L.

**Supplemental Table 2**. The association between clinical parameters and LOS: univariate linear regression

| Variables | group | B (95% CI) | t | P value |
| --- | --- | --- | --- | --- |
| Sex | Male | **0 (ref)** | -- | -- |
|  | Female | -0.86 (-1.18, -0.55) | -5.43 | <0.001 |
| Age, year | 18-45 | **0 (ref)** | -- | -- |
|  | 45-65 | -0.06 (-0.46, 0.34) | -0.28 | 0.779 |
|  | ≥65 | 2.59 (2.20, 2.99) | 12.85 | <0.001 |
| Hospital | Xin Hua Hospital | **0 (ref)** | -- | -- |
|  | Ren Ji Hospital | 2.32 (1.88, 2.77) | 10.28 | <0.001 |
| BMI, kg/m^2^ | <18.5 | -0.12 (-0.36, 0.12) | -0.98 | 0.33 |
|  | 18.5-24 | **0 (ref)** | -- | -- |
|  | ≥24 | 0.00 (-0.13, 1.31) | 0.00 | 0.998 |
| CCI, points | 0 | **0 (ref)** | -- | -- |
|  | 1-2 | 0.16 (-0.16, 0.49) | 0.98 | 0.326 |
|  | ≥3 | 4.42 (3.94, 4.91) | 17.82 | <0.001 |
| Surgery | No | **0 (ref)** | -- | -- |
|  | yes | 1.15 (0.84, 1.45) | 7.34 | <0.001 |
| Vitamin D status | Normal | **0 (ref)** | -- | -- |
|  | Low | 0.45 (0.12, 0.78) | 2.69 | 0.007 |
| Calcium, mmol/L | <2.25 | -0.59 (-0.91, -0.27) | -3.59 | <0.001 |
|  | 2.25-2.75 | **0 (ref)** | -- | -- |
|  | ≥2.25 | 1.63 (0.88, 2.39) | 4.25 | <0.001 |
| Magnesium, mmol/L | <0.75 | 3.76 (3.03, 4.48) | 10.17 | <0.001 |
|  | ≥0.75 | **0 (ref)** | -- | -- |
| Sodium, mmol/L | <135 | 3.99 (3.27, 4.71) | 10.83 | <0.001 |
|  | 135-145 | **0 (ref)** | -- | -- |
|  | ≥145 | 0.73 (0.08, 1.39) | 2.19 | 0.028 |
| Potassium, mmol/L | <3.5 | -0.28 (-0.61, 0.05) | -1.67 | 0.094 |
|  | 3.5-5 | **0 (ref)** | -- | -- |
|  | ≥5 | 2.54 (0.14, 5.24) | 1.85 | 0.064 |
| Chloride, mmol/L | <96 | 5.35 (4.40, 6.31) | 10.97 | <0.001 |
|  | 96-108 | **0 (ref)** | -- | -- |
|  | ≥108 | 1.40 (0.84, 1.96) | 4.89 | <0.001 |
| eGFR-EPI, ml/min/1.73m^2^ | 30-60 | 2.75 (2.23, 3.27) | 10.43 | <0.001 |
|  | 60-90 | 1.96 (1.60, 2.32) | 10.61 | <0.001 |
|  | ≥90 | **0 (ref)** | -- | -- |
| Albumin, g/L | <35 | 4.69 (4.27, 5.11) | 21.84 | <0.001 |
|  | ≥35 | **0 (ref)** | -- | -- |
| Pre-albumin, mg/L | <160 | 5.26 (4.80, 7.71) | 22.45 | <0.001 |
|  | ≥160 | **0 (ref)** | -- | -- |
| Anaemia | No | **0 (ref)** | -- | -- |
|  | Yes | 5.42 (5.04, 5.79) | 28.58 | <0.001 |
| Liver injury | No | **0 (ref)** | -- | -- |
|  | Yes | 0.76 (0.42, 1.09) | 4.39 | <0.001 |
| Dyslipidemia | No | **0 (ref)** | -- | -- |
|  | Yes | 0.49 (0.18, 0.79) | 3.13 | 0.002 |
| FBG, mmol/L | <3.5 | 0.92 (-0.31, 2.15) | 1.46 | 0.143 |
|  | 3.5-7 | **0 (ref)** | -- | -- |
|  | ≥7 | 0.29 (-0.09, 0.66) | 1.51 | 0.131 |
| WBC, ×10^9^/ml | <10 | **0 (ref)** | -- | -- |
|  | ≥10 | 4.52 (3.84, 5.95) | 9.21 | <0.001 |

**Note:**

1. Abbreviation: **LOS**, length of hospital stay; **SD**, standard deviation; **BMI**, body mass index; **CCI,** Charlson Comorbidity index; **eGFR-EPI**, estimated glomerular filtration rate calculated by Chronic Kidney Disease Epidemiology Collaboration equation; **25(OH)D**, 25 hydroxyvitamin D; vitamin **D_3_**_,_ 25 hydroxyvitamin D_3_; vitamin **D_2_**_,_ 25 hydroxyvitamin D_2_; **ALT**, alanine transferase; **AST**, aspartate aminotransferase; **ALP**, alkaline phosphatase; **γ-GT**, gamma glutamyl-transferase**; TBIL,** total bilirubin; **TC**, total cholesterol; **TG**, triglycerides; **LDL-C**, low density lipoprotein cholesterol; **HDL-C**, high density lipoprotein cholesterol; **HIV,** human immunodeficiency virus; **AIDS**, acquired immune deficiency syndrome.
2. Abnormal distribution data was shown as median and quartile range.
3. CCI without terms of HIV infection and AIDS was used to assess the disease severity
4. LOS was defined as the time between the measurement of serum phosphate and discharge time or the time of death.
5. Serum calcium (mmol/L) was calculated as the following equation: serum total calcium (mmol/L) + 0.8×[40-serum albumin (g/L)].
6. Low vitamin D status was defined as serum 25(OH)D level<20 ng/ml or sum of serum vitamin D_3_ and vitamin D_2_ level<20 ng/ml in the absence of 25(OH)D measurement.
7. Liver injury was determined as any of the following: ALT (≥120 U/L), AST (≥150 U/L), ALP (≥250 U/L), **γ-**GT (≥100 U/L), or TBIL (≥34.2 μmol/L).
8. Dyslipidemia was determined if one of the following criteria was met: serum TC ≥6.2 mmol/L, or TG≥2.3 mmol/L, or LDL-C≥4.1 mmol/L, or HDL-C<1.0 mmol/L.
9. Anaemia was determined if serum level of hemoglobin was less than 120 g/L in males, or less than 110 g/L in females.

**Supplemental Table 3**. The association between serum level of phosphate and LOS: subgroup analysis

| Variables | G1 | G2 | | G3 | | G4 | | | G5 | Per 0.1mmol/L decrease  (iP<1.16mmol/L) | Per 0.1mmol/L increase  (iP≥1.16mmol/L) | *P _interaction_* |
| --- | --- | --- | --- | --- | --- | --- | --- | --- | --- | --- | --- | --- |
| Sex | | | | | | | | | | | | 0.146 |
| Males | 4.76 (0.58, 2.96) | | 1.77 (0.58, 2.96) | | 0.34 (-0.16, 0.84) | | **0 (ref)** | -0.08 (-1.1, 0.95) | | 0.64 (0.40, 0.89) | -0.04 (-0.13, 0.05) |  |
| Females | 4.62 (3.16, 6.09) | | 1.52 (0.32, 2.72) | | 0.46 (0.09, 0.82) | | **0 (ref)** | 0.39 (-0.24, 1.02) | | 0.61 (0.40, 0.81) | 0 (-0.08, 0.08) |  |
| Age, years | | | | | | | | | | | | <0.001 |
| 18-45 | 1.72 (0.24, 3.20) | | -0.12 (-1.07, 0.83) | | -0.28 (-0.62, 0.06) | | **0 (ref)** | 0.32 (-0.19, 0.82) | | 0.19 (0.03, 0.36) | 0 (-0.05, 0.07) |  |
| 45-65 | 3.95 (2.22, 5.68) | | 2.09 (0.90, 3.28) | | 0.52 (0.13, 0.92) | | **0 (ref)** | -0.16 (-0.90, 0.57) | | 0.56 (0.34, 0.78) | 0 (-0.09, 0.08) |  |
| ≥65 | 6.55 (4.01, 9.08) | | 2.47 (0.7, 4.24) | | 0.73 (-0.03, 1.5) | | **0 (ref)** | -0.15 (-1.97, 1.67) | | 0.88 (0.54, 1.22) | -0.12 (-0.31, 0.07) |  |
| Hospital |  | |  | |  | |  |  | |  |  | 0.015 |
| Ren Ji | 5.26 (4.11, 6.41) | | 1.93 (1.14, 2.72) | | 0.45 (0.15, 0.74) | | **0 (ref)** | 0.15 (-0.40, 0.70) | | 0.60 (0.45, 0.75) | -0.03 (-0.08, 0.03) |  |
| Xin Hua | 0.50 (-8.01, 7.02) | | 0.76 (-4.2, 5.72) | | 0.58 (-1.37, 2.54) | | **0 (ref)** | -0.50 (-4.82, 3.80) | | 0.65 (-0.38, 1.67) | -0.5 (-1.33, 0.33) |  |
| CCI, points | | | | | | | | | | | | <0.001 |
| 0 | 2.91 (1.36, 4.46) | | 0.93 (-0.08, 0.35) | | -0.03 (-0.42, 0.35) | | **0 (ref)** | 0.40 (-0.23, 1.17) | | 0.39 (0.19, 0.59) | 0.00 (-0.06, 0.06) |  |
| 1-2 | 6.47 (4.34, 8.59) | | 1.47 (-0.02, 2.97) | | 0.52 (-0.03, 1.06) | | **0 (ref)** | 0.01 (-1.02, 1.03) | | 0.71 (0.42, 1.00) | -0.07 (-0.19, 0.06) |  |
| ≥3 | 3.99 (0.12, 7.86) | | 5 (2.27,7.74) | | 1.46 (0.31, 2.61) | | **0 (ref)** | 0.08 (-2.18, 2.34) | | 0.89 (0.35, 1.43) | -0.03 (-0.42, 0.37) |  |
| Albumin, g/L | | | | | | | | | | | | 0.001 |
| <35 | 0.58 (-2.29, 3.45) | | 0.61 (-1.89, 3.09) | | -0.18 (-1.45, 1.08) | | **0 (ref)** | -0.89 (-3.18, 1.40) | | 0.53 (0.07, 0.99) | -0.08 (-0.33, 0.17) |  |
| ≥ 35 | 7.71 (6.12, 9.30) | | 1.92 (0.97, 2.86) | | 0.54 (0.21, 0.87) | | **0 (ref)** | 0.29 (-0.33, 0.90) | | 0.66 (0.47, 0.85) | -0.02 (-0.05 0.04) |  |
| Prealbumin, mg/L | | | | | | | | | | | | <0.001 |
| <160 | 1.6 (-1.10, 4.30) | | 1.76 (-0.66, 4.18) | | 0.84 (-0.53, 2.21) | | **0 (ref)** | 0.46 (-2.06, 2.98) | | 0.54 (0.08, 0.99) | -0.04 (-0.21, 0.13) |  |
| ≥ 160 | 8.03 (6.31, 9.75) | | 1.84 (0.85, 2.82) | | 0.40 (0.07, 0.74) | | **0 (ref)** | 0.03 (-0.60, 0.66) | | 0.68 (0.48, 0.87) | -0.03 (-0.1, 0.04) |  |
| Magnesium, mmol/L | | | | | | | | | | | | 0.055 |
| <0.75 | 7.74 (2.12, 13.37) | | 3.01 (-2.41, 8.43) | | 0.09 (-2.69, 2.88) | | **0 (ref)** | 0.8 (-4.15, 5.76) | | 1.18 (0.42, 1.93) | -0.06 (-1.13, 1.02) |  |
| ≥0.75 | 3.93 (2.54, 5.31) | | 1.69 (0.79, 2.58) | | 0.47 (0.13, 0.80) | | **0 (ref)** | 0.04 (-0.59, 0.66) | | 0.55 (0.37, 0.74) | -0.03 (-0.08, 0.03) |  |
| Vitamin D status | | | | | | | | | | | | <0.001 |
| Normal | 8.72 (5.96, 11.49) | | 1.15 (-0.32, 2.62) | | 0.62 (0.09, 1.16) | | **0 (ref)** | 0.48 (-0.58, 1.54) | | 0.51 (0.23, 0.8) | 0.03 (-0.09, 0.15) |  |
| Low | 3.66 (2.14, 5.18) | | 2.04 (0.93, 3.16) | | 0.32 (-0.12, 0.75) | | **0 (ref)** | 0.07 (-0.71, 0.86) | | 0.64 (0.42, 0.87) | -0.05 (-0.12, 0.03) |  |

**Note**:

1. Abbreviation: **LOS**, length of hospital stay; **SD**, standard deviation; **BMI**, body mass index; **CCI,** Charlson Comorbidity index; **eGFR-EPI**, estimated glomerular filtration rate calculated by Chronic Kidney Disease Epidemiology Collaboration equation; **25(OH)D**, 25 hydroxyvitamin D; vitamin **D_3_**_,_ 25 hydroxyvitamin D_3_; vitamin **D_2_**_,_ 25 hydroxyvitamin D_2_; **ALT**, alanine transferase; **AST**, aspartate aminotransferase; **ALP**, alkaline phosphatase; **γ-GT**, gamma glutamyl-transferase**; TBIL,** total bilirubin; **TC**, total cholesterol; **TG**, triglycerides; **LDL-C**, low density lipoprotein cholesterol; **HDL-C**, high density lipoprotein cholesterol; **HIV,** human immunodeficiency virus; **AIDS**, acquired immune deficiency syndrome.
2. The model was adjusted by sex, age ( “18-45 y”, “45-65 y” ***or*** “≥65 y”), CCI (“0”, “1-2”, ***or*** “≥3”), surgery (“no” ***vs.*** “yes”), hospital (Ren Ji Hospital ***vs.*** Xin Hua hospital), BMI (“<18.5 kg/m^2^”, “18.5-24 kg/m^2^”, ***or*** “≥24 kg/m^2^”), serum level of eGFR-EPI (“30-60 ml/min/1.73m^2^”, “60-90 ml/min/1.73m^2^”, ***or*** “≥90 ml/min/1.73m^2^”), vitamin D status (“normal” ***vs***. “low”), calcium (“<2.25 mmol/L”, “2.25-2.75 mmol/L”, ***or*** “≥2.75 mmol/L”), magnesium (“<0.75 mmol/L” ***vs.*** “≥0.75 mmol/L”), sodium (“<135 mmol/L” , “135-145 mmol/L”, ***or*** “≥145 mmol/L”), chloride (“<96 mmol/L” , “96-108 mmol/L”, ***or*** “≥108 mmol/L”), albumin (“≥35 g/L” ***vs.*** “<35 g/L”), pre-albumin (“≥160 mg/dL” ***vs.*** “<160 mg/dL”), anemia (“no” ***vs.*** “yes”), liver injury (“no” ***vs.*** “yes”), dyslipidemia (“no” ***vs.*** “yes”), and white blood cell count (“<10×10^9^/ml” ***vs.*** “≥10×10^9^/ml”) .
3. CCI without terms of HIV infection and AIDS was used to assess the disease severity
4. LOS was defined as the time between the measurement of serum phosphate and discharge time or the time of death.
5. Serum calcium (mmol/L) was calculated as the following equation: serum total calcium (mmol/L) + 0.8×[40-serum albumin (g/L)].
6. Low vitamin D status was defined as serum 25(OH)D level<20 ng/ml or sum of serum vitamin D_3_ and vitamin D_2_ level<20 ng/ml in the absence of 25(OH)D measurement.
7. Liver injury was determined as any of the following: ALT (≥120 U/L), AST (≥150 U/L), ALP (≥250 U/L), **γ-**GT (≥100 U/L), or TBIL (≥34.2 μmol/L).
8. Dyslipidemia was determined if one of the following criteria was met: serum TC ≥6.2 mmol/L, or TG≥2.3 mmol/L, or LDL-C≥4.1 mmol/L, or HDL-C<1.0 mmol/L.
9. Anaemia was determined if serum level of hemoglobin was less than 120 g/L in males, or less than 110 g/L in females.

**Supplemental Table 4**. The association between serum level of phosphate and LOS using multiple imputed data

| Group | **iP** (mmol/L) | β coefficient (95% CI) | P value | *P trend* |
| --- | --- | --- | --- | --- |
| G1 | <0.64 | 5.07 (3.79, 6.33) | <0.001 | <0.001 |
| G2 | 0.64-0.8 | 1.84 (0.97, 2.70) | <0.001 |  |
| G3 | 0.8-1.16 | 0.42 (0.09, 0.75) | 0.011 |  |
| G4 | 1.16-1.45 | **0 (ref)** | **-** |  |
| G5 | ≥1.45 | 0.17 (-0.44, 0.78) | 0.583 |  |
| Inflection point | 1.16 |  |  | - |
| < inflection point | Per 0.1 mmol/L decrease | 0.65 (0.48, 0.82) | <0.001 | - |
| ≥inflection point | Per 0.1 mmol/L increase | -0.02 (-0.08, 0.04) | 0.513 | - |
| *P for log-likelihood ratio test* | | <0.001 |  |  |

**Note**:

1. Abbreviation: **iP,** inorganic phosphorus; **LOS**, length of hospital stay; **CI**, confidence interval; **BMI**, body mass index; **CCI,** Charlson Comorbidity index; **eGFR-EPI**, estimated glomerular filtration rate calculated by Chronic Kidney Disease Epidemiology Collaboration equation; **25(OH)D**, 25 hydroxyvitamin D; **Vitamin D_3_**_,_ 25 hydroxyvitamin D_3_; **Vitamin D_2_**_,_ 25 hydroxyvitamin D_2_; **ALT**, alanine transferase; **AST**, aspartate aminotransferase; **ALP**, alkaline phosphatase; **γ-GT**, gamma glutamyl-transferase**; TBIL,** total bilirubin; **TC**, total cholesterol; **TG**, triglycerides; **LDL-C**, low density lipoprotein cholesterol; **HDL-C**, high density lipoprotein cholesterol; **HIV,** human immunodeficiency virus; **AIDS**, acquired immune deficiency syndrome.

2. **Fully-adjusted model:** adjusting sex and age (“18-45 y”, “45-65 y” ***or*** “≥65 y”), CCI (“0”, “1-2”, ***or*** “≥3”), surgery (“no” ***vs.*** “yes”), hospital (Ren Ji Hospital ***vs.*** Xin Hua hospital), surgery (“no” ***vs.*** “yes”), BMI (“<18.5 kg/m^2^”, “18.5-24 kg/m^2^”, ***or*** “≥24 kg/m^2^”), serum level of eGFR-EPI (“30-60 ml/min/1.73m^2^”, “60-90 ml/min/1.73m^2^”, ***or*** “≥90 ml/min/1.73m^2^”), vitamin D status (“normal” ***vs***. “low”), calcium (“<2.25 mmol/L”, “2.25-2.75 mmol/L”, ***or*** “≥2.75 mmol/L”), magnesium (“<0.75 mmol/L” ***vs.*** “≥0.75 mmol/L”), sodium (“<135 mmol/L” , “135-145 mmol/L”, ***or*** “≥145 mmol/L”), chloride (“<96 mmol/L” , “96-108 mmol/L”, ***or*** “≥108 mmol/L”), albumin (“≥35 g/L” ***vs.*** “<35 g/L”), pre-albumin (“≥160 mg/dL” ***vs.*** “<160 mg/dL”), anemia (“no” ***vs.*** “yes”), liver injury (“no” ***vs.*** “yes”), dyslipidemia (“no” ***vs.*** “yes”), and white blood cell count (“<10×10^9^/ml” ***vs.*** “≥10×10^9^/ml”) .

4. CCI without terms of HIV infection and AIDS was used to assess the disease severity.

5. LOS was defined as the time between the measurement of serum phosphate and discharge time or the time of death.

6. Serum calcium (mmol/L) was calculated as the following equation: serum total calcium (mmol/L) + 0.8×[40-serum albumin (g/L)].

7. Low vitamin D status was defined as serum 25(OH)D level<20 ng/ml or sum of serum vitamin D_3_ and vitamin D_2_ level<20 ng/ml in the absence of 25(OH)D measurement.

8. Liver injury was determined as any of the following: ALT (≥120 U/L), AST (≥150 U/L), ALP (≥250 U/L), **γ-**GT (≥100 U/L), or TBIL (≥34.2 μmol/L).

9. Dyslipidemia was determined if one of the following criteria was met: serum TC ≥6.2 mmol/L, or TG≥2.3 mmol/L, or LDL-C≥4.1 mmol/L, or HDL-C<1.0 mmol/L.

10. Anemia was determined if serum level of hemoglobin was less than 120 g/L in males, or less than 110 g/L in females.

**Supplemental Table 5**. The association between serum level of phosphate and in-hospital mortality using multiple imputed data

| Group | iP (mmol/L) | Odd ratio (95% CI) | P | *P trend* |
| --- | --- | --- | --- | --- |
| G1 | <0.64 | 3.51 (1.93, 7.04) | <0.001 | 0.004 |
| G2 | 0.64-0.8 | 1.77 (0.88, 3.55) | 0.109 |  |
| G3 | 0.8-1.16 | 0.81 (0.49, 1.33) | 0.400 |  |
| G4 | 1.16-1.45 | **1 (ref)** | **-** |  |
| G5 | ≥1.45 | 1.23 (0.53, 2.86) | 0.633 |  |
| Per 0.1 mmol/L decrease | | 1.12 (1.04, 1.21) | 0.002 |  |

**Note:**

1. Abbreviation: **iP,** inorganic phosphorus; **LOS**, length of hospital stay; **SD**, standard deviation; **BMI**, body mass index; **CCI,** Charlson Comorbidity index; **eGFR-EPI**, estimated glomerular filtration rate calculated by Chronic Kidney Disease Epidemiology Collaboration equation; **25(OH)D**, 25 hydroxyvitamin D; **Vitamin D_3_**_,_ 25 hydroxyvitamin D_3_; **Vitamin D_2_**_,_ 25 hydroxyvitamin D_2_; **ALT**, alanine transferase; **AST**, aspartate aminotransferase; **ALP**, alkaline phosphatase; **γ-GT**, gamma glutamyl-transferase**; TBIL,** total bilirubin; **TC**, total cholesterol; **TG**, triglycerides; **LDL-C**, low density lipoprotein cholesterol; **HDL-C**, high density lipoprotein cholesterol; **HIV,** human immunodeficiency virus; **AIDS**, acquired immune deficiency syndrome.
2. The model was adjusted by sex, age (“18-45 y”, “45-65 y” ***or*** “≥65 y”), CCI (“0”, “1-2”, ***or*** “≥3”), surgery (“no” ***vs.*** “yes”), hospital (Ren Ji Hospital ***vs.*** Xin Hua hospital), LOS (“<7 days”, “7-13 days” ***or***. “≥14 days”), BMI (“<18.5 kg/m^2^”, “18.5-24 kg/m^2^”, ***or*** “≥24 kg/m^2^”), serum level of eGFR-EPI (“30-60 ml/min/1.73m^2^”, “60-90 ml/min/1.73m^2^”, ***or*** “≥90 ml/min/1.73m^2^”), vitamin D status (“normal” ***vs***. “low”), calcium (“<2.25 mmol/L”, “2.25-2.75 mmol/L”, ***or*** “≥2.75 mmol/L”), magnesium (“<0.75 mmol/L” ***vs.*** “≥0.75 mmol/L”), sodium (“<135 mmol/L” , “135-145 mmol/L”, ***or*** “≥145 mmol/L”), chloride (“<96 mmol/L” , “96-108 mmol/L”, ***or*** “≥108 mmol/L”), albumin (“≥35 g/L” ***vs.*** “<35 g/L”), pre-albumin (“≥160 mg/dL” ***vs.*** “<160 mg/dL”), anemia (“no” ***vs.*** “yes”), liver injury (“no” ***vs.*** “yes”), dyslipidemia (“no” ***vs.*** “yes”), fasting blood glucose (“<3.5 mmol/L”, “3.5-7 mmol/L”, ***or*** “≥7 mmol/L”), and white blood cell count (“<10×10^9^/ml” ***vs.*** “≥10×10^9^/ml”) .
3. CCI without terms of HIV infection and AIDS was used to assess the disease severity.
4. LOS was defined as the time between the measurement of serum phosphate and discharge time or the time of death.
5. Serum calcium (mmol/L) was calculated as the following equation: serum total calcium (mmol/L) + 0.8×[40-serum albumin (g/L)].
6. Low vitamin D status was defined as serum 25(OH)D level<20 ng/ml or sum of serum vitamin D_3_ and vitamin D_2_ level<20 ng/ml in the absence of 25(OH)D measurement.
7. Liver injury was determined as any of the following: ALT (≥120 U/L), AST (≥150 U/L), ALP (≥250 U/L), **γ-**GT (≥100 U/L), or TBIL (≥34.2 μmol/L).
8. Dyslipidemia was determined if one of the following criteria was met: serum TC ≥6.2 mmol/L, or TG≥2.3 mmol/L, or LDL-C≥4.1 mmol/L, or HDL-C<1.0 mmol/L.
9. Anemia was determined if serum level of hemoglobin was less than 120 g/L in males, or less than 110 g/L in females.

**Supplemental Figure 1**. Prevalence of phosphate abnormality in in 23,479 Chinese patients


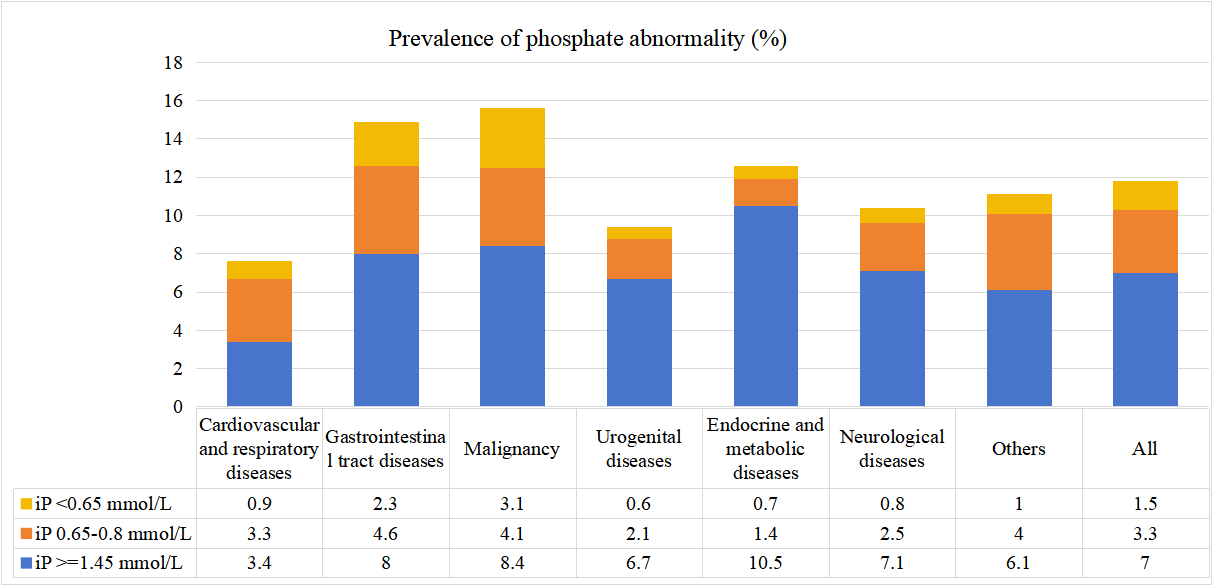


**Supplemental Figure 2**. “L-shaped” non-linear relation between serum phosphate level and length of hospital stay


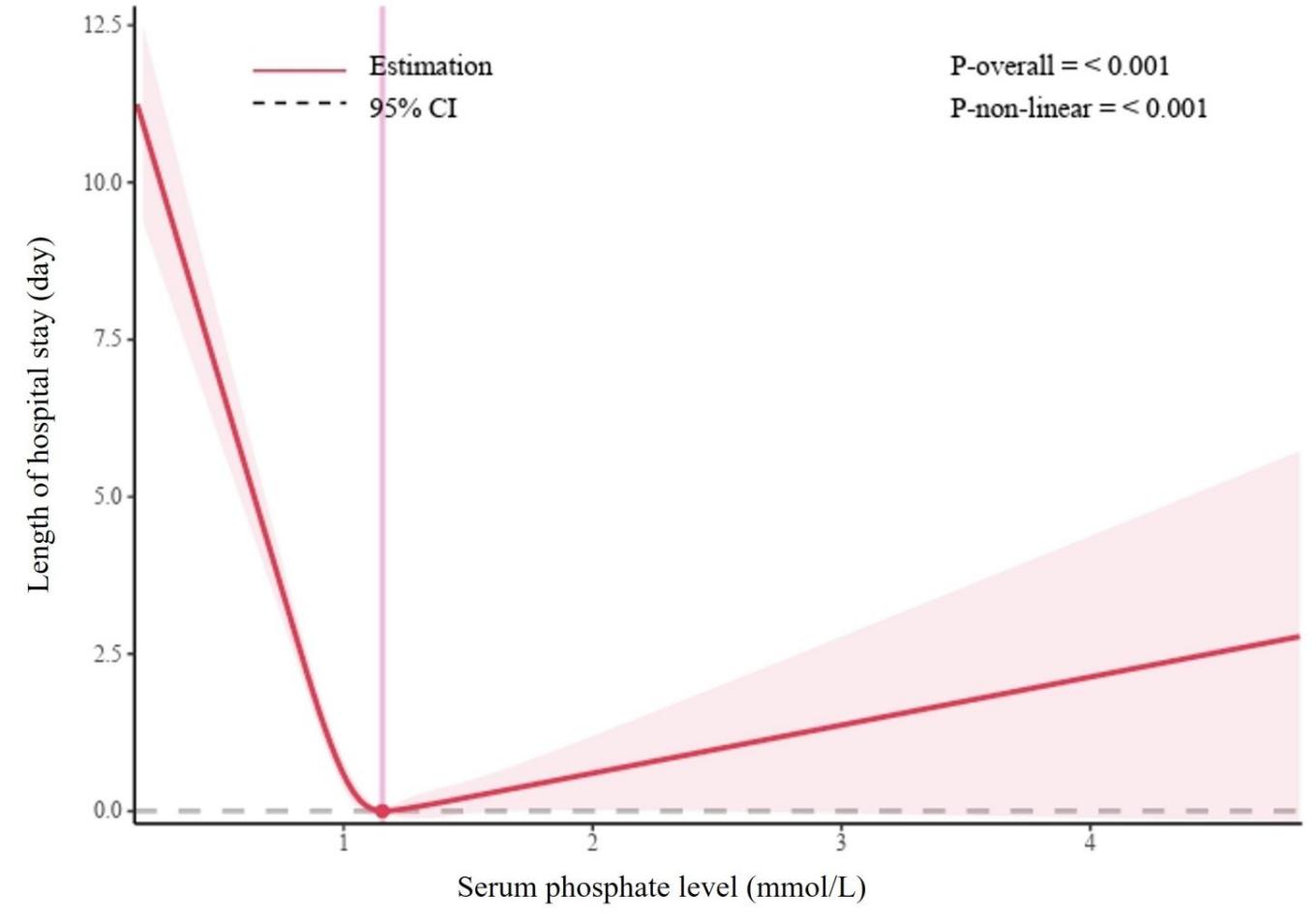


**Notes:**

1. Abbreviation: **LOS**, length of hospital stay; **BMI**, body mass index; **CCI,** Charlson Comorbidity index; **eGFR-EPI**, estimated glomerular filtration rate calculated by Chronic Kidney Disease Epidemiology Collaboration equation; **25(OH)D**, 25 hydroxyvitamin D; **Vitamin D_3_**_,_ 25 hydroxyvitamin D_3_; **Vitamin D_2_**_,_ 25 hydroxyvitamin D_2_; **HIV,** human immunodeficiency virus; **AIDS**, acquired immune deficiency syndrome.
2. Cubic spine analysis adjusted for sex, age ( “18-45 y”, “45-65 y” ***or*** “≥65 y”), CCI (“0”, “1-2”, ***or*** “≥3”), surgery (“no” ***vs.*** “yes”), BMI (“<18.5 kg/m^2^”, “18.5-24 kg/m^2^”, ***or*** “≥24 kg/m^2^”), serum level of eGFR-EPI (“30-60 ml/min/1.73m^2^”, “60-90 ml/min/1.73m^2^”, ***or*** “≥90 ml/min/1.73m^2^”), vitamin D status (“normal” ***vs***. “low”) and calcium (“<2.25 mmol/L”, “2.25-2.75 mmol/L”, ***or*** “≥2.75 mmol/L”).
3. LOS was defined as the time between the measurement of serum phosphate and discharge time or the time of death.
4. CCI without terms of HIV infection and AIDS was used to assess the disease severity
5. Serum calcium (mmol/L) was calculated as the following equation: serum total calcium (mmol/L) + 0.8×[40-serum albumin (g/L)].

Low vitamin D status was defined as serum 25(OH)D level<20 ng/ml or sum of serum vitamin D_2_ and vitamin D_3_ level<10 ng/ml in the absence of 25(OH)D measurement.

**Supplemental Figure 3**. Kaplan-Meier survival analysis of survival weeks in different phosphate categories

**Abbreviation**: **iP**, inorganic phosphorus
